# Biomechanical Effect of Orthodontic Treatment of Canine Retraction by Using Metallic Orthodontic Mini-Implant (OMI) Covered with Various Angles of Revolving Cap

**DOI:** 10.1155/2021/9952392

**Published:** 2021-07-12

**Authors:** Kuson Tuntiwong, Jui-Ting Hsu, Shih-Guang Yang, Jian-Hong Yu, Heng-Li Huang

**Affiliations:** ^1^School of Dentistry, China Medical University, Taichung 40402, Taiwan; ^2^Department of Bioinformatics and Medical Engineering, Asia University, Taichung 41354, Taiwan; ^3^Master Program for Biomedical Engineering, China Medical University, Taichung 40402, Taiwan; ^4^Department of Dentistry, China Medical University Hospital, Taichung 40402, Taiwan

## Abstract

**Objective:**

This study evaluated the biomechanical effects of a metallic orthodontic mini-implant (OMI) covered with various types of angled revolving cap on the peri-OMI bone and the canine periodontal ligament (PDL) by finite element (FE) analyses.

**Materials and Methods:**

Three-dimensional FE models included comprised cortical bone and cancellous bone of the maxilla, and the OMIs were created. The forces (0.98 N) pulled in both the canine hook and the revolving cap, pulling towards each other in both directions as loading conditions. The upper surface of the maxilla was fixed as a boundary condition.

**Results:**

The bone stresses were increasing in the models by using OMI covered with a revolving cap as compared with that in the conventional model (in which only the OMI was placed). However, no obvious differences in bone stresses were observed among the models with various types of angled revolving cap. The minimum principal strain in the canine PDL was highest for condition 180T, followed by condition 180L. However, the maximum differences in the values between each experimental model and the conventional model were around 5%.

**Conclusion:**

This study showed no obvious effects in decreasing or increasing stress/strain in bone and PDL by using various types of angled revolving cap covered metallic mini-implant in orthodontic treatment of canine retraction.

## 1. Introduction

Orthodontic treatment is applied in dentistry for malocclusion, which includes tooth crowding, protrusion, and spacing. Malalignment and crowding are reportedly present in almost 15% of adolescents, and affected adults can have extremely irregular incisors [1]. Tooth extraction is often necessary for treating malalignment and crowding. Two tissues have major effects on orthodontic tooth movement: the periodontal ligament (PDL) and alveolar bone. For cases of Class II or Class I bimaxillary protrusion, normally, the anterior part of the maxillary teeth should be pulled backward and the tooth angulation should be made parallel to the same tooth.

Most cases of orthodontic treatment first involve premolar extraction followed by canine retraction (CR). An anchorage is required for such treatment, which refers to the resistance to reaction forces provided by other teeth, palate, head, or neck (via extraoral forces) [2], and an orthodontic mini-implant (OMI) is commonly used [3]. The ideal force for translating a canine is typically 150–200 gf. The availability of a reliable anchorage during space closure is important when performing CR after premolar extraction. OMIs are suitable for facilitating orthodontic tooth movement due to their biocompatibility, small size, and placement utility [4, 5]. The placement of an OMI between the roots of the maxillary second premolar and first molar for retracting a canine is aimed at avoiding forward movement of the posterior segments of the maxillary arch. However, an OMI can only be placed between teeth with sufficient bone density and root clearance [4].

One limitation of the maxilla is that the maxillary sinus is an important vital structure. The revolving cap can be used with the OMIs and allows forces to be applied to the cap at diverse angulations and is particularly advantageous since an OMI cannot be inserted or position changes by an OMI are greatly restricted in many positions close to vital structures. The revolving cap was constructed from a medical-grade plastic (polycarbonate) material.

The mechanics of orthodontic tooth movement due to remodeling were investigated using finite element (FE) analysis [6]. This technique is popular in orthodontics since it can reveal internal strains and resolve significantly indefinite force systems. [7, 8]. Numerous studies have attempted to improve the modeling of biological structures, with that of Cattaneo et al. probably the most important for different PDL assumptions [7]. Those authors found that making different assumptions about the PDL markedly influences the resulting stress in the ligament. However, modeling approaches are still necessary since *in vivo* studies remain insufficient for investigating biomechanical effects such as stress and strain in PDL.

Therefore, this study used computer-aided design (CAD) and FE analysis to evaluate the effects of various loading systems on the maxillary bone and PDL in cases of CR performed using a revolving cap. The objectives of this study also included determining the effects of applying forces in various directions by angulating the revolving cap and evaluated the maximum and minimum principal strains in the maxillary canine PDL as well as the equivalent (von Mises) stress in the maxillary bones to fully characterize the biomechanics of orthodontic CR.

## 2. Materials and Methods

**Figure 1:**
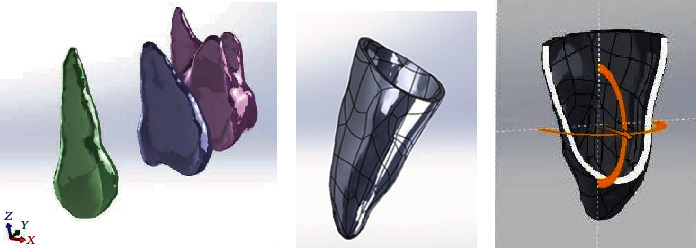
Solid models of the (a) canine, second premolar, and first molar and (b) canine PDL. (c) The PDF was 0.25 mm thick.

A series of computed tomography images of the maxilla of an orthodontic patient was obtained from the left upper teeth (canine, second premolar, and first molar) and PDL (Somatom Sensation 16, Siemens Medical Solutions, Forchheim, Germany). Adjacent images were separated by 1 mm. The coordinate data were used by CAD software (SolidWorks 2007, SolidWorks, Concord, MA, USA) to generate a three-dimensional solid model of the left maxilla. The PDL tissue was modeled as a thin enclosure around the dental root with an average thickness of approximately 0.25 mm [9] (Figure 1). The cortical bone was modeled as being 2 mm thick, with underlying cancellous bone. The maxilla was cut in half and had manipulated alveolar sockets, and a model of the tooth was also created.

A model of a 10 mm long OMI with a diameter of 1.6 mm was constructed manually using CAD software (SolidWorks 2007). The OMI was angled 60° from the alveolar ridge between the roots of the second premolar and the first molar on the buccal side, and it was positioned 1 mm from the PDL. This location eliminated interference from adjacent roots and was based on the local root anatomy. A subtraction operation was performed to create a hole for the OMI in the maxillary model. The 0.018 in standard nontorqued, nonangulated, edgewise orthodontic canine brackets; molar tube; OMI; and revolving cap were modeled manually based on a typical clinical treatment process. The reconstructed maxilla, canine, second premolar, first molar, PDL, brackets, OMI, and revolving cap were all modeled using CAD software (SolidWorks 2007) (Table 1).

**Figure 2:**
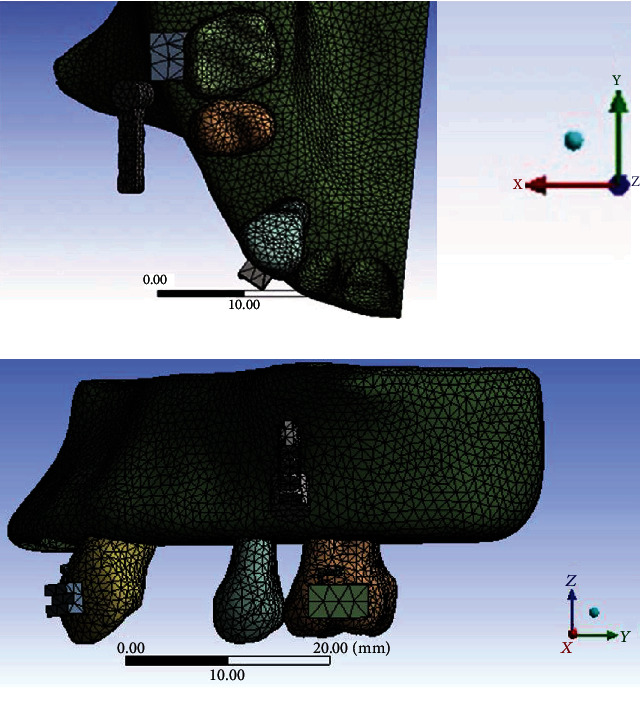
(a) The upper view and (b) frontal view of the FE model in this study.

**Figure 3:**
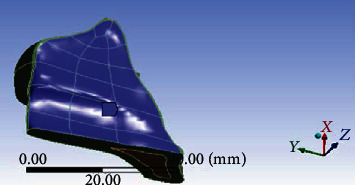
The boundary condition set at the upper surface of the maxilla.

All models were combined using Boolean operations, and files containing the models in IGES format were then imported into ANSYS Workbench (Swanson Analysis, Huston, PA, USA) to generate the FE model (Figure 2) using 10-node tetrahedral *h*-elements. Homologous, isotropic, and linearly elastic material properties were used for all models except for the PDL, which was included as a bilinear elastic material [10–13]. The material properties assigned to the FE models are listed in Table 2. In the simplified model, a 0.98 N (100 gf) couple force was applied as the loading condition either towards the canine hook or towards the revolving cap (Table 1). The mesiodistal surfaces of the maxilla bone were constrained to zero displacement in the *x*, *y*, and *z* directions as a boundary condition. A fixed boundary condition assigned to the upper sectioned surface of the maxillary bone as shown in Figure 3 completed the simulation of natural anatomic constraints.

An FE analysis produces an approximate rather than an exact solution. Therefore, the convergence of the FE models was tested to verify the mesh quality, with a maximum element size of 2.0 mm set for meshing in all FE models used in this study. Orthodontic forces were simulated according to normal clinical practice. The loading conditions consisted of the following different angulations of the revolving cap that was placed to cover the head of OMI: 0°, 45°, 90°, 135°, and 180°. A traction force was applied at two locations on the cap for each of these angles: (1) at the top of the cap (“T” suffix) or low down the cap (“L” suffix). Hence, a total of 10 sets of FE models was analyzed: 0T, 0L, 45T, 45L, 90T, 90L, 135T, 135L, 180T, and 180L (Table 1).

## 3. Results

### 3.1. Equivalent (von Mises) Stress in the Maxilla

**Figure 4:**
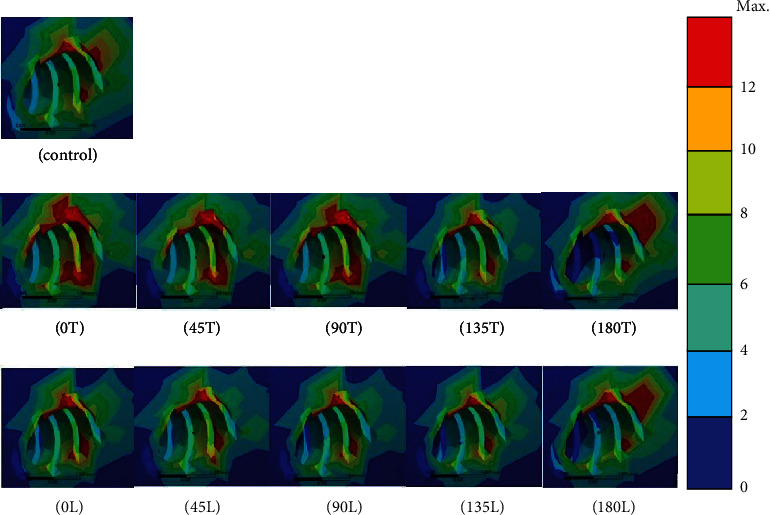
von Mises stress distributions for CR in the conventional model (control) and the experimental models. Areas with high stresses are indicated in red.

Distinct stresses were evident in the cortical bone only in the peri-OMI bone and not in the adjacent areas around the socket of the teeth. The results indicate that condition 0T (37.6 MPa) induced the highest equivalent (von Mises) stress in the bone. Moreover, conditions 180L (34.1 MPa), 90T (32.9 MPa), 180T (32.2 MPa), 45T (28.9 MPa), 0 L (27.4 MPa), 135L (23.5 MPa), 45L (23.4 MPa), 135T (23.1 MPa), and 90L (22.5 MPa) are implied. The stress was lowest in the conventional model (22.1 MPa). The areas with high compressive stresses (indicated as red areas in the figures) around the uppermost OMI thread hole were much larger in conditions 180T and 180L than in the conventional model. No marked differences in the stresses were observed between the conventional model and conditions 0L, 45L, 90L, 135T, and 135L (Figure 4).

### 3.2. Maximum Principal Elastic Strain in the Canine PDL

**Figure 5:**
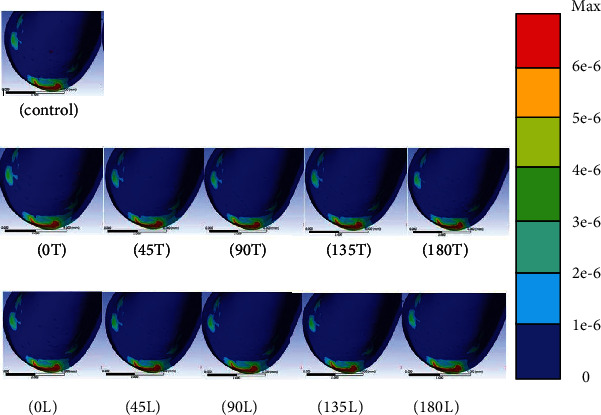
Distributions of the maximum principal strain for CR in the conventional model (control) and the experimental models. Areas with high tensile strains are indicated in red. (*e*‐6 indicates 1∗10^−6^).

The principal strain in the canine PDL was highest when traction forces were applied. Regions exhibiting compressive and tensile normal strains could be identified in the PDL. However, the magnitude of the tensile strain was significantly higher than that of the compressive strain. All dominant principal strains were clearly evident at the mesiolabial margin of the PDL, with no marked differences in the strain distribution between the models (Figure 5). The strain was the highest in condition 0L (14.9 *μ*strain), followed by conditions 0T (14.8 *μ*strain), 45L and 90L (14.7 *μ*strain), and 45T, 90T, 135T, and 135L (14.6 *μ*strain). The maximum strain in the conventional model was 14.3 *μ*strain. The peak strain was lower in conditions 180L (14.2 *μ*strain) and 180T (13.9 *μ*strain) than in the conventional model. The difference in the values between each experimental model and the conventional model did not exceed 5%.

### 3.3. Minimum Principal Elastic Strain in the Canine PDL

**Figure 6:**
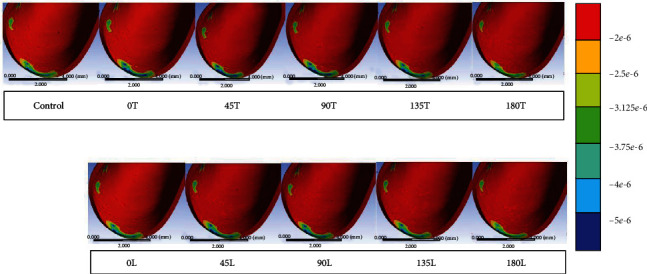
Distributions of the minimum principal strain for CR in the conventional model (control) and the experimental models. Areas with high compressive strains are indicated in blue. (*e*‐6 presents 1∗10^−6^).

The principal strain in the canine PDL was the lowest when compressive forces were applied. An area of compressive strain was clearly evident at the mesiolabial margin of the PDL and on the distal surface of the inner PDL. The minimum strain peaked at condition 180T (−7.5 *μ*strain), followed by condition 180L (−7.6 *μ*strain); both of these values were higher than those in the conventional model. The minimum strain was −7.9 *μ*strain for conditions 0L, 45L, and 90L and −7.8 *μ*strain for conditions 0T, 90T, 135T, and 135L. The minimum strain in the conventional model and condition 45T was −7.7 *μ*strain. When translation occurred, an area of compression started to appear on the distal surface of the inner PDL in the direction of the applied force. A blue area appeared on the distal surface of the inner PDL for conditions 180T and 180L, which was almost perfectly aligned with the initial canine translation (Figure 6). The maximum differences in the values between each experimental model and the conventional group were around 5%.

## 4. Discussion

OMIs are often used to induce tooth translation in the dental clinic. This study has introduced the innovation of utilizing a revolving cap to overcome some of the limitations associated with using an OMI. The biomechanical performance when using various orthodontic loading directions with an OMI and a revolving cap anchorage in the case of orthodontic CR was investigated. The maximum and minimum principal strains in the canine PDL were examined to determine differences in CR. The von Mises stress was examined in the peri-OMI cortical bone [14], which may be related to the risk of bone resorption. A revolving cap is a supplementary device used in CR to overcome limitations in the positioning of the OMI.

This study found that the values of tensile strain (maximum principal strain) in the canine PDL in all models did not differ by more than 5% from those in the conventional model when a revolving cap was located on the OMI between the second premolar and the first molar. During orthodontic treatment, the revolving cap can be easily managed in cases of maxillary pneumatization. Moreover, the minimum principal strain is usually a compressive strain, and the compression at the canine PDL margin was on the inner mesiolabial surface, which indicates the occurrence of tipping. The minimum principal strain in the canine PDL for an angulation of 180° relative to the revolving cap resulted in the desired orthodontic movement.

The von Mises stresses in the peri-OMI bone of the maxilla was highest for condition 0T (37.6 MPa) and varied between 22 and 34 MPa in the conventional model as well as in the other experimental models when the revolving cap was located on the OMI between the second premolar and the first molar. Stress values from 34 to 48 MPa will induce bone resorption, following the studies of Qian et al. and Frost, which means using a revolving cap in condition 0T is not recommended [15, 16].

There is considerable interest among both patients and orthodontists in using OMIs to move teeth due to their advantages over both conventional methods and the use of extraoral devices. It is relatively straightforward to use a revolving cap to cover the OMI to facilitate tooth movement and hence also the translational mechanics phase of orthodontic treatment. This study showed that covering mini-implants by a revolving cap increased the peri-OMI bone stress. In addition to the risk of pathologic bone resorption, there is also a possibility of crestal bone resorption based on the high stress values induced in the peri-OMI bone when using the revolving cap under an angulation of 0°. This is probably due to the distance from the original force to the revolving cap area, which would result in higher bone stress. Fortunately, a revolving cap at an angulation of 0° is not used frequently in orthodontic treatments.

This study was subject to a variety of limitations, including the inherent ones that apply in any modeling study. A model can only produce results as accurate as the set of assumptions that were used to create it, including boundary conditions, loading conditions, and material properties. The boundary conditions of the present model included that the top surface of the maxilla was fixed. The loading conditions included applying load over the revolving cap covering OMI, which was placed between the second premolar and the first molar sites. The position of the OMI was set between the root of the second premolar and the first molar. Therefore, the present findings might not apply to other positions of OMIs and revolving caps.

## 5. Conclusions

Within the limitations as described above, the findings of this study can be summarized as follows:

1. No obvious effects in decreasing bone stress were observed in all models by using mini-implant (OMI) covered with various angles of the revolving cap. However, the stress around the top of the OMI thread hole was greatly increased in conditions 180T and 180L compared with the conventional model. No obvious differences in the stress in the peri-OMI bone were found between the conventional model and conditions 0L, 45L, 90L, 135T, and 135L
2. All dominant principal strains were clearly evident at the mesiolabial margin of the PDL. However, the highest strain in each experimental model did not differ by more than 5% from that in the conventional model. It looks like there are no obvious effects in decreasing or increasing the strain in PDL by using the revolving cap in orthodontic treatment of canine retraction

## Figures and Tables

**Table 1:** The simulations involved ten models with different positions of the revolving cap and loading types (red arrows).

Model	Position of force	Loading condition
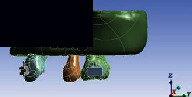	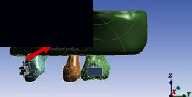	0T
	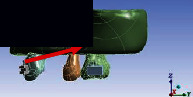	0L

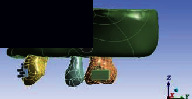	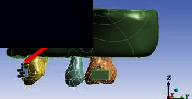	45T
	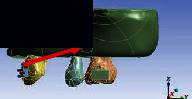	45L

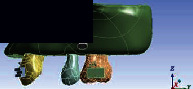	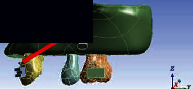	90T
	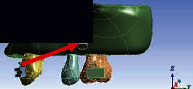	90L

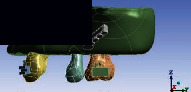	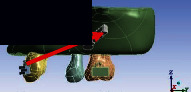	135T
	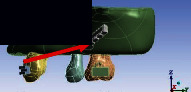	135L

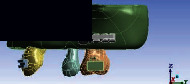	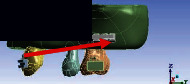	180T
	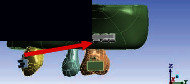	180L

**Table 2:** Material properties used in the FE model. [10–13].

Material	Young's modulus (MPa)	Poisson's ratio
Tooth	1.8 × 10^4^	0.30
PDL (bilinear)	*E*_1_ = 0.15 *E*_2_ = 0.6	0.30
Cancellous bone	460	0.30
Cortical bone	1.49 × 10^4^	0.30
Bracket and tube	2.3 × 10^5^	0.30
OMI (stainless steel)	2.3 × 10^5^	0.30
Revolving cap (polycarbonate)	2.75 × 10^3^	0.38

## Data Availability

The underlying data can be found through the authors. Anybody who is interested in the data can email the authors directly.

## References

[1] Proffit W., Field H. (1993). *Malocclusion and dentofacial deformity in contemporary society, in Contemporary Orthodontics*.

[2] Roberts-Harry D., Sandy J. (2004). Orthodontics. Part 9: anchorage control and distal movement. *British Dental Journal*.

[3] Park H.-S., Bae S.-M., Kyung H.-M., Sung J.-H. (2001). Micro-implant anchorage for treatment of skeletal class I bialveolar protrusion. *Journal of Clinical Orthodontics*.

[4] Ammar H. H., Ngan P., Crout R. J., Mucino V. H., Mukdadi O. M. (2011). Three-dimensional modeling and finite element analysis in treatment planning for orthodontic tooth movement. *American Journal of Orthodontics and Dentofacial Orthopedics*.

[5] Papadopoulos M. A., Tarawneh F. (2007). The use of miniscrew implants for temporary skeletal anchorage in orthodontics: a comprehensive review. *Oral Surgery, Oral Medicine, Oral Pathology, Oral Radiology, and Endodontics*.

[6] Mohammed S., Desai H. (2014). Basic concepts of finite element analysis and its applications in dentistry: an overview. *Journal of Oral Hygiene & Health*.

[7] Cattaneo P., Dalstra M., Melsen B. (2005). The finite element method: a tool to study orthodontic tooth movement. *Journal of Dental Research*.

[8] Poppe M., Bourauel C., Jäger A. (2002). Determination of the elasticity parameters of the human periodontal ligament and the location of the center of resistance of single-rooted teeth. *Journal of Orofacial Orthopedics*.

[9] Coolidge E. D. (1937). The thickness of the human periodontal membrane. *Journal of the American Dental Association (1939)*.

[10] Liao Z., Chen J., Li W., Darendeliler M. A., Swain M., Li Q. (2016). Biomechanical investigation into the role of the periodontal ligament in optimising orthodontic force: a finite element case study. *Archives of Oral Biology*.

[11] Kawarizadeh A., Bourauel C., Jäger A. (2003). Experimental and numerical determination of initial tooth mobility and material properties of the periodontal ligament in rat molar specimens. *European Journal of Orthodontics*.

[12] Lin T.-S., Tsai F.-D., Chen C.-Y., Lin L.-W. (2013). Factorial analysis of variables affecting bone stress adjacent to the orthodontic anchorage mini-implant with finite element analysis. *American Journal of Orthodontics and Dentofacial Orthopedics*.

[13] Lande M. S. M., Desai A., Verma A., Gaur A. (2015). Stress analysis of polycarbonate spur gears for sugarcane juice machine using FEA. *International Journal on Recent Technologies in Mechanical and Electrical Engineering (IJRMEE)*.

[14] Motoyoshi M., Ueno S., Okazaki K., Shimizu N. (2009). Bone stress for a mini-implant close to the roots of adjacent teeth - 3D finite element analysis. *International Journal of Oral and Maxillofacial Surgery*.

[15] Qian L., Todo M., Matsushita Y., Koyano K. (2009). Finite element analysis of bone resorption around dental implant. *Journal of Biomechanical Science and Engineering*.

[16] Frost H. M. (1992). Perspectives: bone's mechanical usage windows. *Bone and Mineral*.

